# Chloride-liberal fluids are associated with acute kidney injury after liver transplantation

**DOI:** 10.1186/s13054-014-0625-7

**Published:** 2014-11-19

**Authors:** Ashraf Nadeem, Nawal Salahuddin, Alyaa El Hazmi, Mini Joseph, Balsam Bohlega, Hend Sallam, Yasser Sheikh, Dieter Broering

**Affiliations:** Department of Adult Critical Care Medicine, King Faisal Specialist Hospital and Research Centre, At Takhassusi, Al Madhar Ash Shamali, Riyadh 12713 Saudi Arabia; Department of Nursing Services, King Faisal Specialist Hospital and Research Centre, At Takhassusi, Al Madhar Ash Shamali, Riyadh 12713 Saudi Arabia; Organ transplant Centre, King Faisal Specialist Hospital and Research Centre, At Takhassusi, Al Madhar Ash Shamali, Riyadh 12713 Saudi Arabia

## Abstract

**Introduction:**

Acute kidney injury (AKI) occurs frequently after liver transplantation and is associated with significant morbidity and mortality. Recent evidence has linked the predominant usage of ‘chloride-liberal’ intravenous fluids, such as 0.9% saline to the development of renal dysfunction in general critically ill patients. We compared the effects of perioperative fluid types on AKI in liver transplant recipients.

**Methods:**

An observational analysis of liver transplant recipients over a 33-month period, between January 2010 and September 2013, was performed. Intensive care unit database and patient records were analyzed for determinants of early postoperative AKI. Univariate and multivariate regression analysis was carried out using a two-tailed *P* value less than 0.05 to establish significance. The institutional Research Ethics Committee approved the study methodology (RAC no. 2131 073).

**Results:**

One hundred and fifty-eight liver transplants were performed, AKI developed in 57 (36.1%) patients: 39 (68.4%) fully recovered, 13 (22.8%) developed chronic renal failure and 10 (17.5%) required long-term hemodialysis. On univariate regression analysis, AKI was significantly associated with greater than 3,200 ml of chloride-liberal fluids infused within the first postoperative day (HR 5.9, 95% CI 2.64, 13.2, *P* <0.001), greater than 1,500 ml colloids received in the operating room (hazard ratio (HR) 1.97, 95% CI 1.01, 3.8, *P* = 0.046), vasopressor requirement for 48 hours posttransplant (HR 3.34, 95% CI 1.55, 7.21, *P* = 0.002), hyperchloremia at day 2 (HR 1.09, 95% CI 1.01, 1.18, *P* = 0.015) and preoperative model for end-stage liver disease (MELD) score (HR 1.08, 95% CI 1.03, 1.13, *P* <0.001).

After stepwise multivariate regression, infusion of greater than 3,200 ml of chloride-liberal fluids (HR 6.25, 95% CI 2.69, 14.5, *P* <0.000) and preoperative MELD score (HR 1.08, 95% CI 1.02, 1.15, *P* = 0.004) remained significant predictors for AKI.

**Conclusions:**

In a sample of liver transplant recipients, infusion of higher volumes of chloride-liberal fluids and preoperative status was associated with an increased risk for postoperative AKI.

## Introduction

Acute kidney injury (AKI) occurs both frequently after liver transplantation, reportedly in 29 to 60% recipients [1-3] and, irrespective of severity, confers an increased risk of death [4]. This increase in risk of mortality extends from the early postoperative period (28 days) and up to one year after transplantation [1]. The National Institute of Diabetes and Digestive and Kidney Disease (NIDDK) long-term follow-up study ascribed a 2.66 hazard ratio (HR) directly attributable to renal dysfunction developing after liver transplantation [5].

Previously described risk factors for AKI in liver recipients are greater severity of illness pretransplant (higher model for end-stage liver disease (MELD) scores, intensive care unit (ICU) admission, and coagulopathy), vasopressors, and greater transfusions in the immediate perioperative period [5-13]. However, very little is known about the effects of intravenous fluid selection (chloride-liberal versus chloride-restrictive) or the effects of fluid balance on the risk of renal injury. An overall fluid overload state leads to renal congestion, compromised renal blood flow and reductions in glomerular filtration rate (GFR) [14]. In critically ill patients, a positive fluid balance has been associated with increased mortality [15,16] and poorer outcomes once AKI develops [17-19]. Recent evidence has highlighted the possible nephrotoxic effects of ‘chloride-liberal’ fluids (0.9% saline). Animal and human controlled studies have shown that infusions of chloride-liberal or solutions with supraphysiological chloride concentrations cause vasoconstriction of renal afferent arterioles, cortical hypoperfusion and decreased GFR [20-22]. In a recent pre- and postintervention study, restriction to chloride-restrictive fluids was associated with lower AKI and need for renal replacement therapy (RRT) as compared to chloride-liberal fluids [23]. Therefore, perioperative intravenous fluid selection and volume may prove to be a modifiable risk factor for the prevention of AKI. No similar data exists for liver transplant recipients. These patients clearly are at an increased risk of renal injury with mortal consequences. We hypothesized that AKI occurring in the early postoperative period after liver transplant may be associated with the use of chloride-liberal fluids and overall fluid overload causing renal congestion.

## Material and methods

This study is reported following the STROBE statement checklist for observational studies [24]. All studies at our institution require ethical approval; the Office of Research Affairs (ORA) and ORA Research Ethics Committee approved the study (RAC no. 2131 073). Patient consent was waived by the Research Ethics Committee.

### Study design and setting

This was an observational study of liver transplant recipients carried out at a tertiary care, university hospital over a 33-month period between January 2010 and September 2013.

### Operational definitions

AKI was defined according to the risk, injury, failure, loss, end-stage renal failure (RIFLE) classification [25] of renal dysfunction, that is using both increases in creatinine from preoperative values and urine output measured as urine volume in milliliters/patient’s baseline weight in kilograms/hour. Serum creatinine values were measured preoperatively and daily for up to the third postoperative day. Serum creatinine was measured using the COBAS Integra Creatinine plus ver. 2 assay (Roche Diagnostics Corp, Basel, Switzerland). This is an enzymatic method based on the determination of hydrogen peroxide after conversion of creatinine with the aid of creatininase, creatinase, and sarcosine oxidase. Patients were screened for the development of postoperative AKI on a daily basis until the third day.

Prolonged ICU stay was defined *a priori* as a cutoff value of the mean (or median for skewed data) ICU days after transplantation. Delayed weaning from mechanical ventilation was defined as <3 > days of invasive ventilation.

Chloride-liberal fluids were fluids containing supraphysiological concentrations of chloride (0.9% saline, 20% and 5% albumin); chloride-restrictive fluids were fluids with chloride concentrations closer to plasma (0.45% saline, Ringer’s lactate).

### Participants

Consecutive adult liver transplant recipients within the specified study period were included. Patients undergoing multiorgan transplantation were excluded.

Crystalloids used in the study patients were: lactated Ringer’s (sodium chloride, potassium chloride, sodium lactate and calcium chloride) injection, 0.9% sodium chloride injection, USP, 0.45% sodium chloride injection, USP, manufactured by Baxter Healthcare Corp, Deerfield IL, USA. Colloids used were: human albumin 5% and 20% manufactured by Biotest Pharma GmbH, Dreieich, Germany.

### Variables

The primary outcome variable was the development of postoperative AKI*.* Other outcome variables studied were delayed weaning from mechanical ventilation, prolonged ICU stay *(*as defined above), ICU mortality, and 28-day mortality. Other variables collected were recipient demographic data, etiology of cirrhosis, comorbidities, posttransplant acute physiologic and chronic health evaluation II (APACHE II) scores, routine hematological, biochemical and organ dysfunction/physiological (AKI, vasopressors, RRT, mechanical ventilation) data, fluid balance, fluids and blood products received at admission to ICU and daily up to day 3 posttransplantation.

### Data sources/measurement

In our ICU, patient data is routinely entered into an ICU database. Data entry is by a critical care nurse dedicated to the database. Patient data required for the study and missing from the database was extracted by the research team (AH, HS, BB, AN) from the patient’s electronic medical records and laboratory computerized results.

### Statistical analysis

Continuous data was tested for normality: measures of central tendency were compared as means ± standard deviations (SD) using the Student’s *t* test for normally distributed variables and as medians (interquartile range, IQR) using the Mann-Whitney *U* test for skewed data. Categorical variables were compared using the chi-square test or the Fisher exact test for n <5. Fluid volumes were dealt with as continuous variables while fluid types were classified into either ‘chloride-liberal’ or ‘chloride-restrictive’ and correspondingly dealt with as continuous variables. Logistic regression analysis was performed to determine the predictive ability of variables for AKI, prolonged ICU stay and prolonged mechanical ventilation. Univariate and multivariate techniques were used, and for multivariate regression, a backward mode with a threshold 0.15 was used for elimination. Multivariate associations were reported as odds ratios (OR) with 95% confidence intervals (CIs). A two-sided *P* value of <0.05 was considered as statistically significant. All analyses were carried out using IBM SPSS version 22.0 (IBM Corp, Armonk, NY, USA).

## Results

### Participants and descriptive data

One hundred and fifty-eight liver transplants were performed during the study period. Of these, 104 (65.8%) were living donor-related transplants, 53 (33.5%) cadaveric and 1 (0.6%) was a retransplant. Mean age of transplant recipients was 52.3 ± 13.3 years and 66 (42%) were female, mean body mass index (BMI) was 26.8 ± 6 (range 14, 49). Mean MELD score at time of transplantation was 19.4 ± 7.7 (range 6, 45), with mean baseline creatinine 88.7 micromol/L ± 56 (range 27, 350). Transplant recipients had end-stage liver disease caused by: hepatitis C in 60 (38%) patients, hepatitis B in 34 (21.5%), cryptogenic liver disease in 41 (25.9%), autoimmune disease in 9 (5.7%) and others (bilharziasis, congenital hepatic cirrhosis, Budd-Chiari, Wilson’s disease) in 14 (9%) recipients. Fifty-two (32.9%) patients had hepatocellular carcinomas.

**Figure 1:**
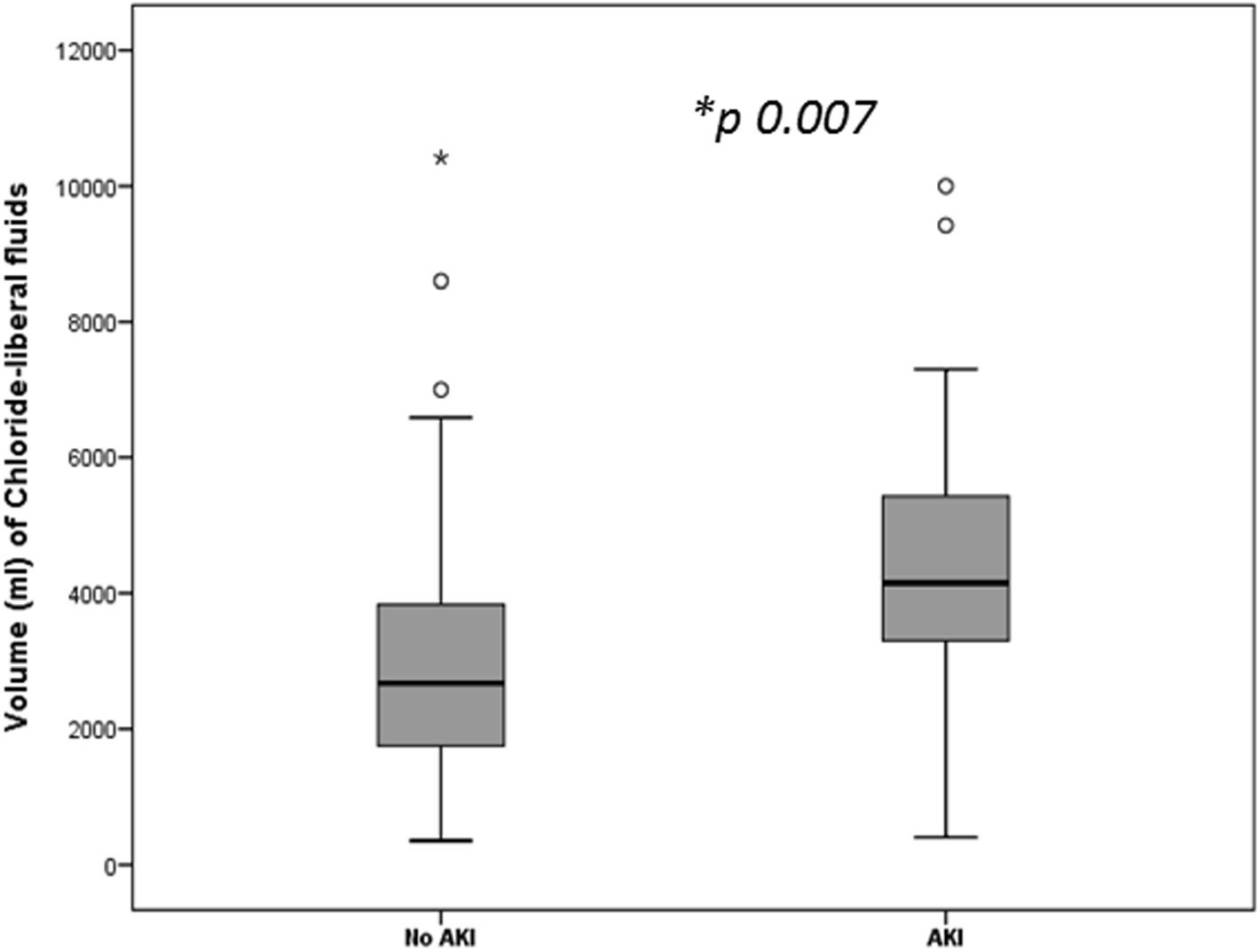
**Relative volumes of chloride-liberal fluids received by liver transplant recipients with and without acute kidney injury (AKI).**

All patients were transferred to the ICU posttransplant. On arrival, transplant recipients were in a positive fluid balance of 8.48 ± 2.3 liters fluid, having received 1.7 ± 0.26 L packed red cells, 3.8 ± 0.143 L blood products (plasma, cryoprecipitate, platelets) and a mean of 7.8 ± 6.3 L crystalloids of which 5.6 ± 4.0 L were chloride-liberal and 2.4 ± 1.4 L were chloride-restrictive fluids. Mean APACHE II score was 15.9 ± 5.4 (range 4, 48), serum procalcitonin level 2.4 ngm/ml (IQR 3.1), proBNP 476 pg/mL (IQR 2,510) and 120 (76%) patients were on a norepinephrine infusion. Seventy-eight (49.4%) had pleural effusions; pleural drainage by pigtail catheters was carried out in 23 out of 78 patients (29.4%). In the first 72 hours of ICU stay, transplant recipients were in a cumulative positive fluid balance of 12.7 ± 7.8 L with 9 ± 6 L crystalloids, 6.4 ± 3.5 L colloids (5%, 20% albumin) and 25 ± 11.9 L chloride-liberal (0.9% saline, 5% albumin, 20% albumin) received. Blood products transfused were 4 L (IQR 4.8). There was no significant difference between the mean serum chloride levels in the chloride-liberal group compared to the chloride-restrictive group, 114 ± 5.8 versus 113 ± 5.9, *P* >0.05. No significant correlation was found between the mean serum chloride concentrations and the cumulative volumes of chloride-liberal fluids given, *P* >0.05 (see Table 1 and Figure 1).

**Table 1:** **Chemical compositions of fluids used in liver transplant recipients**

***Fluid-type***	***Composition per 1 liter***	***Manufacturer***
***crystalloids***		
0.9% Sodium chloride, USP	154 mEq Sodium	Baxter Healthcare Corp, Deerfield, IL, USA
	154 mEq Chloride	
0.45% Sodium chloride, USP	77 mEq Sodium	Baxter Healthcare Corp, Deerfield, IL, USA
	77 mEq Chloride	
Lactated Ringer’s injection, USP	130 mEq Sodium	Baxter Healthcare Corp, Deerfield, IL, USA
	4 mEq Potassium	
	3 mEq Calcium	
	109 mEq Chloride	
	28 mEq Sodium lactate	
***Colloids***		
Human albumin 5% biotest	Plasma protein 50gm (96% albumin), caprylate (4 mmol/l), N-acetyl-DL-tryptophanate (4 mmol/l), sodium ions (145 mmol/l), water for injections ad 1,000 ml	Biotest Pharma GmbH
		Dreieich, Germany
Human albumin 20% biotest (given diluted in 0.9% normal saline)	200 g/l (at least 95% is human albumin)	Biotest Pharma GmbH
		Dreieich, Germany

Median ICU length of stay was five days (IQR 6), mean 8.4 ± 12.5 days. Patients were ventilated for a median of two days (IQR 2) with nine (5.7%) patients subsequently undergoing tracheostomy. ICU mortality was 8.2% (13 patients); 28-day survival was 140 (88.6%). Eight (5%) recipients were readmitted to the ICU for the following: respiratory failure two, sepsis three, bleeding three. Mean time to readmission was 3.5 ± 1.1 days (2, 5).

### Outcomes

AKI developed in 58 (36.4%) patients; classified as risk in 30 (52%), injury in 12 (21%) and failure in 16 (27%) patients. Ninety percent (53 patients) developed AKI within the first two postoperative days. All liver transplant patients, both the group that developed AKI and those that did not were on the calcineurin inhibitor, tacrolimus. Drug levels were routinely monitored and dosing adjusted accordingly. Thirty-nine (68.4%) patients recovered fully, 13 (22.8%) developed chronic renal failure and 10 (17.5%) required long-term hemodialysis. AKI was significantly associated with ICU mortality, *P* = 0.001 and 28-day mortality, *P* <0.001. Mean serum chloride levels on the second postoperative day were significantly greater in patients who developed AKI compared to those who did not; 114 ± 7.2 versus 112 ± 4.4, *P =* 0.01. The mean chloride levels on days one and three were not significantly different. There were no significant associations between mean serum chloride levels and the severity of renal failure (see Table 2).

**Table 2:** **Characteristics of liver transplant patients grouped by acute kidney injury according to the RIFLE classification**

	**No AKI** **(n = 101)**	**AKI** **(n =57)**	***P*** **value**
***Age***, *years*	51 ± 14.2	54 ± 11.3	0.13
***BMI***	26.4 ± 6	27.7 ± 6.6	0.19
***Pretransplant creatinine***, *micromol/L*	76.2 ± 40.2	110.8 ± 72.6	0.001
***Pretransplant MELD score***	19.2 ± 5.4	21.8 ± 9.1	0.001
***Volume of chloride-liberal fluids***, *liters (IQR)*			
Operating room	4.7 (5.7)	6 (6.8)	0.23
24 hours	2.1 (1.2)	3.8 (2.7)	<0.001
48 hours	0.64 (1.06)	1.7 (1.5)	0.007
72 hours	0.35 (0.65)	1.07 (0.87)	0.55
***Volume of chloride-restrictive fluids***, *liters (IQR)*			
Operating room	2 (2)	2 (1.2)	0.46
24 hours	1.9 (1.28)	1.8 (1.4)	0.32
48 hours	2.0 (0.76)	1.9 (1.6)	0.86
72 hours	1.3 (1.0)	1.6 (1.1)	0.67
***Volume of colloids***, *liters (IQR)*			
Operating room	1.5 (1)	2.1 (2.5)	0.013
24 hours posttransplant	1.2 (1.03)	1.9 (1.4)	0.001
48 hours posttransplant	0.45 (0.85)	0.95 (0.71)	0.016
72 hours posttransplant	0.35 (0.65)	1.0 (0.87)	0.05
***Volume of packed RBC transfusions***, *liters (IQR)*			
Operating room	1.5 (1.2)	2.2 (1.9)	0.022
24 hours	0.76 (0.44)	1.14 (5.8)	0.19
48 hours	0.53 (0.38)	0.72 (0.53)	0.08
72 hours	0.38 (0.71)	0.38 (0.06)	0.89
***Volume of blood products transfused***, *liters (IQR)*			
Operating room	3.2 (2.3)	3.8 (3.2)	0.17
24 hours	0.95 (2.1)	2.1 (4.8)	0.14
48 hours posttransplant	0.72 (0.53)	0.97 (1.2)	0.63
72 hours posttransplant	0.63 (1.5)	0.51 (0.32)	0.78
***Volume of crystalloids infused***, *liters (IQR)*			
Operating room	6 (5.6)	6 (6)	0.8
24 hours	2.5 (1.3)	3.1 (1.9)	0.15
48 hours	2.1 (0.72)	2.4 (1.4)	0.22
72 hours	1.6 (0.96)	1.7 (1.1)	0.30
***Fluid balance***, *liters (IQR)*			
Operating room	6.4 (4.7)	6.7 (5.4)	0.76
24 hours	2.9 (2.6)	3.7 (4.8)	0.09
48 hours	1.6 (1.5)	1.7 (2.4)	0.06
72 hours	0.947 (2.07)	0.623 (1.54)	0.93
***Serum lactate***, mmol/L			
Day 1	4.2 ± 2.9	4.8 ± 3.9	0.26
Day 2	1.8 ± 1.1	2.1 ± 1.6	0.09
Day 3	1.4 ± 0.5	1.9 ± 0.3	0.046
***Serum sodium***, mmol/L			
Day 1	146 ± 5.2	146.5 ± 5.0	0.57
Day 2	143 ± 5.7	145 ± 3	0.016
Day 3	144 ± 2.3	146 ± 2.6	0.002
***Serum chloride***, mmol/L			
Day 1	114 ± 5.7	113 ± 5.9	0.76
Day 2	112 ± 4.4	114 ± 7.2	0.01
Day 3	110 ± 4.6	111 ± 4.8	0.18
***Vasopressor requirement***			
At admission	73 (72%)	47 (82.5%)	0.25
Day 1	3 (3%)	1 (1.8%)	0.64
Day 2	15 (14.8%)	21 (36.8%)	0.002
Day 3	4 (4%)	8 (14%)	0.056
***Days on mechanical ventilation***	2.3 ± 3.6	6 ± 7.1	0.001
***Length of ICU stay after transplant***	5.5 ± 4.7	13.4 ± 19	0.003

AKI, acute kidney injury; BMI, body mass index; ICU, intensive care unit; IQR, interquartile range; MELD, model for end-stage liver disease; RIFLE, risk, injury, failure, loss, end-stage renal failure.

### Univariate outcome data

On univariate regression analysis, AKI was significantly associated with greater than 3,200 ml of chloride-liberal fluids infused within the first postoperative day (HR 5.9, 95% CI 2.64, 13.2, *P* <0.001), greater than 1,500 ml colloids received in the OR (HR 1.97, 95% CI 1.01, 3.8, *P* = 0.046), vasopressor requirement for 48 hours posttransplant (HR 3.34, 95% CI 1.55, 7.21, *P* = 0.002), hyperchloremia at day 2 (HR 1.09, 95% CI 1.01, 1.18, *P* = 0.015) and preoperative MELD score (HR 1.08, 95% CI 1.03, 1.13, *P* = 0.001).

Delayed weaning from mechanical ventilation was associated with higher volumes of chloride-liberal fluids, *P* = 0.02, higher colloid volumes, *P* = 0.015, blood products transfused, *P* = 0.017 and a cumulative positive fluid balance, *P* = 0.026. Higher pretransplant MELD scores, *P* = 0.001, male gender, *P* = 0.015, transplant for hepatocellular carcinoma, *P* = 0.014, crystalloid volume received in the first 72 hours, *P* = 0.034, need for vasopressors at 48 hours, *P* <0.001 and 72 hours, *P* = 0.031, AKI, *P* <0.001 and pleural effusion, *P* = 0.001 were significantly associated with a prolonged ICU admission. Drainage of effusion was significantly associated with a reduced ICU stay, *P* = 0.007 (see Tables 3 and 4).

**Table 3:** **Demographic, fluids and outcome variables in liver transplant patients grouped by ICU length of stay**

	**<5 days ICU stay (n = 78)**	**≥5 days ICU stay (n = 80)**	***P*** **value**
***Male gender***	25 (32%)	41 (51.2%)	0.015
***HCC***	33 (42.3%)	19 (23.1.7%)	0.014
***Pretransplant MELD score***	17 ± 5.9	21.3 ± 8	0.001
***Volume of crystalloids received by 72 hours*** *ml (IQR)*	1,675 (1,055)	1,520 (1,066)	0.034
***Volume of colloids received by*** *ml (IQR)*			
24 hours posttransplant	1,247 (1,263)	1,700 (1,188)	0.046
48 hours posttransplant	650 (713)	950 (1095)	0.015
***Undrained pleural effusion posttransplant***	28 (35.8%)	50 (62.5%)	0.001
***Vasopressors requirement***			
48 hours posttransplant	8 (10.3%)	28 (35%)	0.001
72 hours posttransplant	2 (2.6%)	10 (12.5%)	0.031
***AKI***	17 (22%)	40 (50%)	<0.001
***Early complications***	43 (55.1%)	50 (62.5%)	0.035

AKI, acute kidney injury; HCC, hepatocellular carcinoma; ICU, intensive care unit; IQR, interquartile range; MELD, model for end-stage liver disease.

**Table 4:** **Perioperative fluids in patients after liver transplant grouped by delayed weaning from mechanical ventilation**

	**<3 days mechanical ventilation (n = 104)**	**≥3 days mechanical ventilation (n = 54)**	***P*** **value**
***Volume of blood products***,^*^ *ml (IQR)*			
Operating room	4,030 (2391)	5,321 (4607)	0.015
24 hours posttransplant	920 (1551)	3,028 (3893)	0.017
48 hours posttransplant	415 (470)	827 (1194)	0.048
***Volume of colloids***, *ml (IQR)*			
Operating room	1,500 (1875)	2,421 (850)	0.032
24 hours posttransplant	1,842 (1019)	2,315 (1205)	<0.001
48 hours posttransplant	625 (669)	1,193 (933)	<0.001
72 hours posttransplant	675 (881)	700 (883)	0.015
***Volume of chloride-liberal fluids***, *ml (IQR)*			
Operating room	5,000 (7813)	7,000 (8434)	0.027
24 hours posttransplant	3,397 (2868)	3,725 (2473)	0.020
***Fluid balance at 48 hours posttransplant***, *ml*	1,725 (1186)	2,257 (2102)	0.026

^*^Includes packed cells, fresh frozen plasma, platelets, cryoprecipitate. IQR, interquartile range.

### Multivariate analysis

After adjusting for covariates, infusion of greater than 3,200 ml of chloride-liberal fluids (HR 6.25, 95% CI 2.69, 14.5, *P* <0.001) and preoperative MELD score (HR 1.08, 95% CI 1.02, 1.15, *P* = 0.004) remained significant predictors for AKI. Prolonged ICU stay was predicted by male gender, *P* = 0.014, vasopressors = 0.003 and the development of AKI, *P* = 0.013 (see Table 5).

**Table 5:** **Regression analysis for variables associated with acute kidney injury post-liver transplantation**

	**Hazard ratio**	**95% CI**	***P*** **value**
***Univariate analysis***			
MELD score	1.08	1.03,1013	0.001
APACHE II score at admission to ICU	1.08	1.03,1.15	0.018
Colloids ≥1,500 ml received in OR	1.97	1.01,3.8	0.046
Chloride-liberal fluids ≥3,200 ml received within the first 24 hours posttransplant	5.9	2.64,13.2	0.000
Vasopressors requirement at 2 days posttransplant	3.34	1.55,7.21	0.002
Serum chloride level at day 2	1.09	1.01,1.18	0.015
***Multivariate analysis***			
Chloride-liberal fluids ≥3,200 ml received within the first 24 hours posttransplant	6.25	2.69,14.5	<0.001
Preoperative MELD score	1.08	1.02,1.15	0.004

APACHE II, acute physiology and chronic health evaluation II; CI, confidence interval; ICU, intensive care unit; MELD, model for end-stage liver disease; OR, operating room.

## Discussion

In this observational study, we found that liver transplant recipients were more likely to develop AKI if they received larger volumes of chloride-liberal (hyperchloremic) fluids. This association was significant, after adjusting for baseline variables, for both 5% albumin in 0.9% saline and only 0.9% saline infusions. Patients who developed AKI had significantly higher serum chloride levels compared to transplant recipients that did not develop AKI.

‘Normal’ saline or 0.9% saline contains supraphysiological levels of chloride (154 mmol/L as compared to Hartmann’s solution, Ringer’s lactate or Plasma-Lyte 148, all of which contain chloride concentrations that are lower (94 to 111 mmol/L). Five percent albumin is available either as salt-poor or in sodium chloride (chloride concentration 128 mmol/L). Intravenous infusions of chloride-liberal fluids have been associated with hyperchloremia and metabolic acidosis when administered in large volumes [26].

Our results show a detrimental effect on renal function with use of chloride-liberal fluids in the immediate postoperative period (up to 48 hours). Support for our findings comes from animal studies that have demonstrated reductions in GFRs, renal arteriolar vasoconstriction [27], and human volunteer studies that have shown reduced renal cortical tissue perfusion, renal blood flow velocity after infusions of hyperchloremic solutions. [28]. In controlled trials, chloride-liberal fluids compared to chloride-poor fluids have been linked to longer time to micturition [21], lower urine output [29] and in a recent observational study of over 31,000 postoperative patients, 0.9% saline compared to ‘balanced’ crystalloids increased the risk of acute renal failure requiring dialysis [30]. Yunos *et al*., in a pre- and postintervention study on 1,530 critically ill patients found that a chloride-restrictive fluid strategy resulted in a significant reduction in AKI, need for RRT and increase in creatinine as compared to a control group given chloride-liberal fluids [23].

Possible explanations for this renal ‘toxicity’ of chloride-liberal fluids come from animal studies that have demonstrated renal vasoconstriction [22] and thromboxane release after chloride infusions [20]. Chloride infusions increase delivery to the macula densa that stimulates glomerulotubular feedback leading to afferent arteriole constriction, mesangial contraction and resultant decrease in GFR [31].

Patients in our study received both 5% albumin in saline and 20% albumin in saline. Though it is possible that the observed renal dysfunction resulted from hyperoncotic albumin, the data on 20% albumin is so far inconclusive. A recent cohort study on 1,000 patients found a higher risk of renal injury and failure with the use of hyperoncotic albumin (OR 5.99) [32], however, a contradictory result was reported from two meta-analyses that concluded no harmful effects of hyperoncotic resuscitation [33,34]. The SAFE study that compared albumin and saline found no difference in adverse outcomes [35].

Fluid overload leads to organ dysfunction due to interstitial edema and visceromegaly. The limited accommodative capacity of the encapsulated kidney causes increased interstitial hydrostatic pressures with reduced renal perfusion and filtration [36]. Additionally, a fluid overload state contributes to third spacing, ascitic fluid accumulation and abdominal compartment syndrome [37]. Cumulative fluid overload has been linked to poor outcomes in all groups (pediatric, septic, postoperative) of critically ill patients with prolonged days on mechanical ventilation, ICU stay and mortality [16,17,38-43]. Recovery of renal function in patients on RRT is also determined by overall fluid balance [17,38,42]. In our study, a cumulative positive fluid balance increased the duration of stay in ICU.

A limitation of our study is that the observational design does not establish a causal relationship of hyperchloremic fluid excess with the development of AKI in liver transplant recipients. These associations may be subject to bias from selection, confounding or random error. We attempted to control for confounders by using regression analysis. Another limitation is the external validity or generalizability of our results to other liver transplant recipients since we collected data only from a single institution.

## Conclusions

In summary, large infusions of chloride-liberal fluids may predict a higher risk of AKI in liver transplant recipients. Our findings support the hypothesis that ‘routine’ intravenous fluids may not be routine and in themselves be associated with organ dysfunction. Our results can be used to build hypotheses for further controlled trials.

## Key messages

- Chloride-liberal fluids may cause renal dysfunction
- Large volumes (>3,200 ml) of chloride-liberal fluids infused in the first 24 hours after liver transplantation were associated with a higher risk of AKI.

## Abbreviations

AKI: acute kidney injury
APACHE II: acute physiologic and chronic health evaluation II
BMI: body mass index
CI: confidence interval
GFR: glomerular filtration rate
HR: hazard ratio
ICU: intensive care unit
IQR: interquartile range
MELD: model for end-stage liver disease
OR: odds ratio
RIFLE: risk, injury, failure, loss, end-stage renal failure
RRT: renal replacement therapy
SD: standard deviation
